# Clinical Trial Assessing the Efficacy of Gabapentin Plus B Complex (B1/B12) versus Pregabalin for Treating Painful Diabetic Neuropathy

**DOI:** 10.1155/2016/4078695

**Published:** 2016-01-17

**Authors:** Alberto Mimenza Alvarado, Sara Aguilar Navarro

**Affiliations:** Geriatrics Department, National Institute of Medical Sciences and Nutrition Salvador Zubirán, Vasco de Quiroga No. 15, Colonia Section XVI, Delegación Tlalpan, 14000 Mexico, DF, Mexico

## Abstract

*Introduction*. Painful diabetic neuropathy (PDN) is a prevalent and impairing disorder. The objective of this study was to show the efficacy and safety of gabapentin (GBP) plus complex B vitamins: thiamine (B1) and cyanocobalamine (B12) compared to pregabalin in patients with moderate to severe intensity PDN. *Method*. Multicenter, randomized, blind study. Two hundred and seventy patients were evaluated, 147 with GBP/B1/B12 and 123 with PGB, with a 7/10 pain intensity on the Visual Analog Scale (VAS). Five visits (12 weeks) were scheduled. The GBP/B1 (100 mg)/B12 (20 mg) group started with 300 mg at visit 1 to 3600 mg at visit 5. The PGB group started with 75 mg/d at visit 1 to 600 mg/d at visit 5. Different safety and efficacy scales were applied, as well as adverse event assessment. *Results*. Both drugs showed reduction of pain intensity, without significant statistical difference (*P* = 0.900). In the GBP/B1/B12 group, an improvement of at least 30% on VAS correlated to a 900 mg/d dose, compared with PGB 300 mg/d. Likewise, occurrence of vertigo was lower in the GBP/B1-B12 group, with a significant statistical difference, *P* = 0.014. *Conclusions*. Our study shows that GPB/B1-B12 combination is as effective as PGB. Nonetheless, pain intensity reduction is achieved with 50% of the minimum required gabapentin dose alone (800 to 1600 mg/d) in classic NDD trials. Less vertigo and dizziness occurrence was also observed in the GBP/B1/B12 group. This trial is registered with ClinicalTrials.gov NCT01364298.

## 1. Introduction

The most common cause of neuropathy worldwide is diabetes mellitus [1]. A neuropathy prevalence of 30% is reported in diabetic patients, estimating more than 50% could suffer from it during the course of the disease [2].

Painful diabetic neuropathy (PDN) is one of the most common causes of chronic pain. Chronic pain affects 30% of the United States (US) population and has high treatment costs, estimated approximately to be 650 billion dollars [3].

Chronic pain treatment requires a multidisciplinary intervention and, sometimes, use of multimodal treatments [3]. This situation has required using combination drugs as a treatment alternative, towards improving the patient prognosis.

There is evidence suggesting that more than half of chronic pain patients receive two or more analgesics, although evidence supporting most of these combinations is limited [4].

Even when efforts for developing new drugs have allowed new treatment options, searching for further alternatives, effective and safe, is necessary. Therefore, it is possible, through synergy between drugs with different mechanisms of action, to provide greater pain killing effects with less adverse events.

Treatment of painful diabetic neuropathy (PDN) includes using of antidepressants, anticonvulsants (calcium channel blockers), and opioid drugs, among others. One of the main problems when using these drugs is adverse events (AE), occasionally limiting the possibility to use drugs recommended in clinical trials [5].

Complex B vitamins, specifically thiamine (B1) and cyanocobalamin (B12), have been shown to be of clinical use in some painful diseases, derived from their effects on the central nervous system, synthesis, and secretion of serotonin in several brain areas [6], blocking metabolic pathways related to oxidative stress [7], as well as their effects on the nitric oxide/guanosine monophosphate cyclic (NO/GMPc) pathway [8], among other mechanisms. Synergy of these vitamins with other drugs, for example, gabapentin, allows for reducing recommended doses of these vitamins as monotherapy, achieving greater reduction effects on pain intensity with less AE occurrence. Gabapentin (GBP), a calcium channel a2*δ* ligand, has proven useful in the treatment of neuropathic pain, with effective results on a daily dosage interval of 1800–3600 mg, although this doses are related to a higher AE rate (nausea, vomit, dizziness, and somnolence of 20–50%) [9]. Pregabalin (PGB), another calcium channel a2*δ* ligand, has also shown benefit in the treatment of neuropathic pain, although such benefits are related to high doses, which are evidently associated with AE occurrence, including dizziness, somnolence, and peripheral edema[10].

Our study objective was to determine the efficacy of gabapentin/vitamins B1 and B12 (GBP/B1/B12) versus pregabalin (PGB) for painful diabetic neuropathy (PDN) during 12 weeks of treatment.

## 2. Materials and Methods

Phase IV, multicenter, randomized, open-label, parallel group, noninferiority study was conducted in Mexico City.

Patients enrolled had the following characteristics:

- Low to moderate intensity PDN.
- Diagnosed by Leeds Assessment of Neuropathic Symptoms and Signs (LANSS).
- ≥1 year of evolution.
- Less than 5 years of being diagnosed.
- Stable hypoglycemic treatment (≥6 weeks).
- In stable condition (HbA1c ≤ 10% at selection visit).
- >40 mm score in the Visual Analog Scale (VAS).
- Numeric Pain Intensity (NPI) Scale (at least 4 days a week) completed on a daily basis during the week previous to randomization.
  - Daily average score of at least 4, during the 7 days prior to randomization.

Subject eligibility was initially assessed in a preselection period of 4–7 days. The selection period was planned to last at least 4 days, to a maximum of 7 days; in this stage, inclusion and exclusion criteria of every subject were assessed according to protocol specifications. At this stage, patients entered a wash-out period equivalent to 3 mean lives of the drug or a maximum of 7 days (whatever happened first) and randomized to either one of the study groups.

The treatment and follow-up stage comprise 6 visits (visit 0 to visit 5), from day 0 to day 84, and a total duration of 12 weeks.

The primary efficacy endpoint was mean change score in VAS. We compared two treatment groups and used a design capable of detecting 0.1-point differences, with a type I error of 0.05 and a power of 0.90, considering a standard deviation (SD) of 26 mm. According to calculation, 286 patients were needed. In order to consider a dropout rate of 25%, a total of 360 patients were considered, 180 in each group.

We used randomization envelopes to control treatment allocation. The randomization list was generated by a statistical program. Randomization was controlled in blocks of 6 patients to achieve a 1 : 1 proportion in the two arms.

We used an ANCOVA analysis with treatment in the model and baseline mean NPI score, as covariates. Differences between treatment groups were assessed each visit, based on adjusted treatment means. The same analysis was done for the VAS. Parametric (paired* t*-tests) and nonparametric (Wilcoxon) statistical methods were applied to compare each visit with baseline and each consecutive visit. Responses of 30% and 50% were analyzed through Pearson chi-squared, on the case of NPI and VAS. Response to the PGIC and CGIC and the time it took the patients to fall asleep were analyzed through a nonparametric method, Gamma statistics.

For other secondary scores resulting from adding several items, as subjective well-being items and profile of mood states factors, comparison between treatments was done by Mann-Whitney tests. We also obtained adjusted ANCOVA means, by baseline measures, of profile of mood states factors, as well as total scores, and tested differences in means. Descriptive statistics of measures were obtained by visit and treatment in general. The analysis considered a last observation carried forward (LOCF) imputation and a type I error of 0.05.

The study was conducted in 270 subjects, 18 to 65 years old, with diabetes mellitus type 1 or 2, and documented diagnosis of sensory motor PDN, moderate to severe, in accordance with LANSS scale [11], and fulfilling the following criteria:

- Neuropathic pain present during at least a year before the study.
- >40 mm score in the Visual Analog Scale (VAS) [12] (at screening and baseline visit).
- Stable hypoglycemic treatment for at least 6 weeks before randomization.
- HbA1c < 8.5% at screening visit.

One group (*n* = 147) received oral gabapentin tablets, 300 mg/thiamine 100 mg/cyanocobalamin 0.20 mg, starting with 300 mg/day (day 1), followed by 900 mg/day on visit 1, 1800 mg/day on visit 2, 2700 mg/day on visit 3, and 3600 mg/day on visits 4 and 5. Other group (*n* = 123) received oral pregabalin capsules, 75 mg/day every 12 h, followed by 300 mg/day every 12 h on visit 2, and followed by 600 mg/day on visits 3, 4, and 5.

In case of patient intolerance upon dose increase in the corresponding visit, patients were kept for the rest of the study with the previous tolerated dose.

We used VAS, Clinical Global Impression (CGI) [13], and Patients' Global Impression of Change (PGIC) [14], at baseline and end of study, to assess pain improvement. Information about sleeping hours overnight was obtained and question 4 of the sleep questionnaire (Mexican population) [15] was analyzed, consisting of 10 questions.

**Figure 1:**
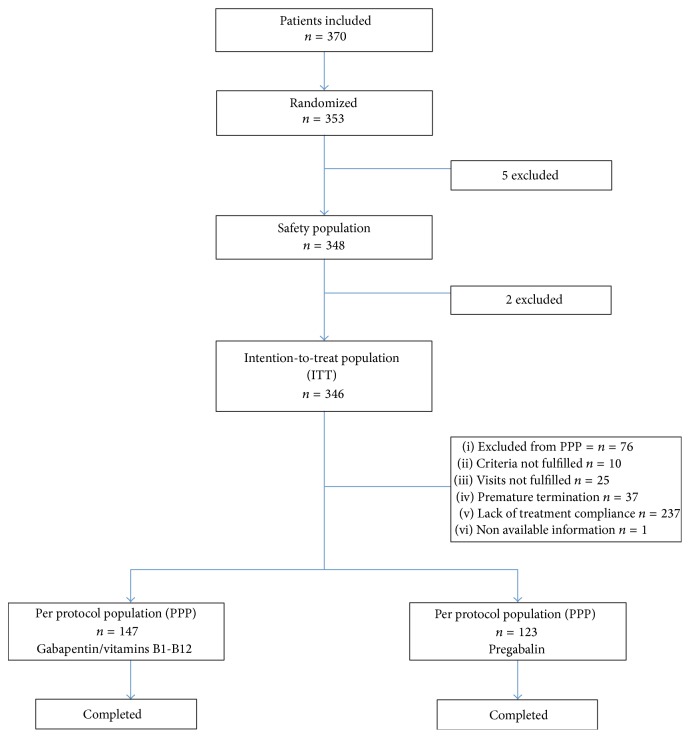
Study design.

We established 5 visits; total study duration was 15 weeks (1 for prescreening, 1 for screening, and 12 weeks of randomized treatment) (see Figure 1).

For the statistical analysis, we assessed homogeneity between groups, applying chi-square for categorical variables and Student's* t*-test for continuous variables. For analyzing changes in baseline and postbaseline changes, as well as between visits, Student's* t*-test was used for matched samples and the Mantel-Haenszel test for safety measures between visits. All statistics tests have a significance level of 0.05 and 95% confidence intervals (CI). We used SPSS software for Windows (SPSS Inc., Chicago, IL, 18,0 version).

*All related and nonrelated adverse events (AE) were recorded*, as well as changes in physical examination (weight and size) and laboratory analysis (including glycated hemoglobin).

The protocol was submitted to and approved by an Independent Ethics Committee in Mexico City, fulfilling all ethics regulations, in accordance with the World Medical Association Declaration of Helsinki of 1975 (Ethical principles for medical research involving human subjects) and 2000 revision. All patients included in the study signed an informed consent to participate in the study.

## 3. Results

Four hundred and fifty nine subjects were selected, 353 of which were randomized; 346 constituted the intention-to-treat population. Five patients had type 1 diabetes (2 in the group of GBP and two in the group of PGB). They were divided in parallel groups: 173 patients treated with GBP/B1/B12 and 173 patients treated with PGB. Two patients were discontinued from the study due to missing information after their initial visit (one of each group), remaining 346 (intention-to-treat population, ITT). Seventy-two patients were discontinued from the study due to several reasons, remaining 270 patients, as per protocol population (PPP) (see Figure 1).

## 4. Sociodemographic Characteristics

Sixty-eight percent of GBP/B1-B12 and 74% of PGB groups were female; average age was 54 (± 9.4 years old) in the PGB group and 53 (± 10.5 years old) in the GBP/B1-B12 group. No significant statistical differences were observed between both groups regarding comorbidity, body mass index (BMI), and glycated hemoglobin (HbA1c) levels; 115 (78) patients in the GBP/B complex used metformin, and 95 (77%) used metformin in the pregabalin group. Diabetes duration, PDN, and treatment used for controlling the disease, as well as other comorbidities, are shown in Table 1.

### 4.1. Efficacy (VAS)

**Figure 2:**
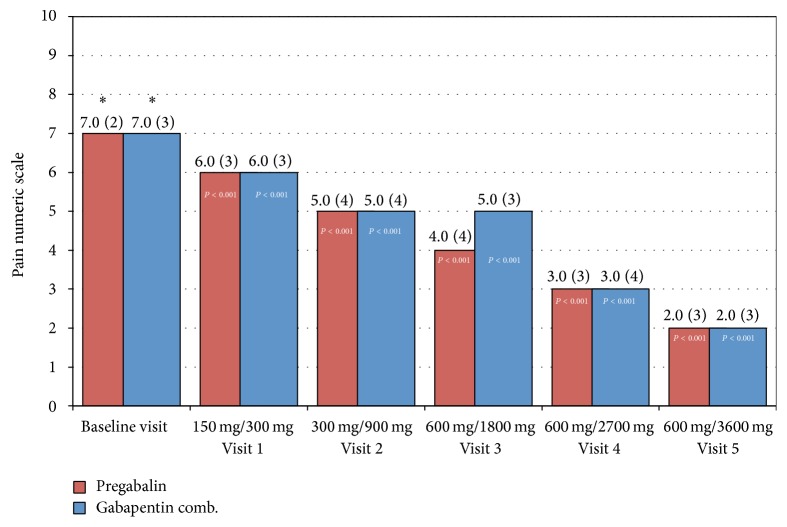
Median comparison per visit, between gabapentin-B complex and pregabalin, in pain intensity reduction, per visit and dose. Pain Visual Analog Scale (VAS), median (interquartile range) per visit, per protocol population. ^**∗**^Statistically significant change from baseline visit, *P* < 0.05. HbA1c: glycated hemoglobin, BMI: body mass index.

Pain intensity at baseline visit was 7 (SD ± 1.5), measured by VAS, in the GBP/B1-B12 group, and 7.1 (SD ± 1.7) in the PGB group. By analyzing pain intensity reduction through VAS, expressed as median, by visit and dose, both drugs equally decreased pain, without a significant statistical difference between both treatment groups, *P* > 0.05. However, pain intensity showed a statistically significant reduction from baseline by visit in both treatment groups, *P* ≤ 0.001 (see Figure 2).

Pain intensity reduction (at least 30%) was 78% for the GBP/B1-B12 group and 85% for PGB, without statistical difference (*P* = 0.133). For a decrease of at least 50%, no significant statistical difference was observed.

### 4.2. Effects on Sleeping

Analysis on improvement of sleep patterns was measured by sleep questionnaire, showing that both drugs improved sleeping hours toward the end of the study, from baseline visit (7.2 h for PGB, *P* = 0.0002, and 7.0 h for GBP, *P* < 0.001). Regarding the sleep questionnaire question, “have you slept all you needed?,” an average change for visit 5 of −0.57 was observed for the GBP/B1-B12 (*P* = 0.0015) group and −0.37 for the PGB (*P* = 0.049) group.

### 4.3. Patients' Global Impression of Change (IGCP)

**Figure 3:**
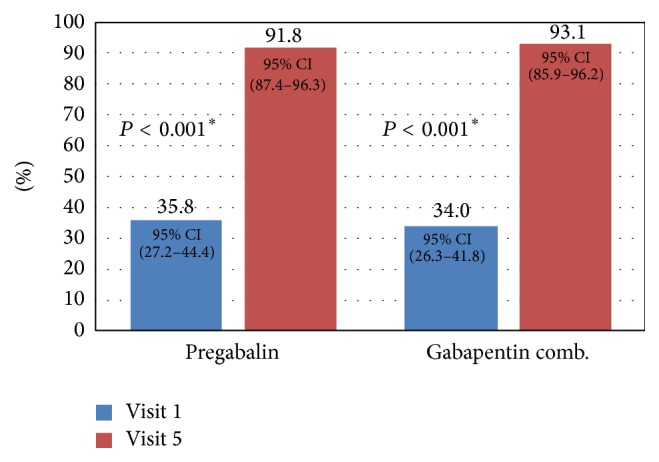
Patients' Global Impression of Change (PGIC) between baseline and visit 5, specifically the question: “From study start, my health is much improved or very much improved.” Patient Global Impression of Change (IGCP) at visit 1 (baseline) and visit 5 (Day 84). From study start, my health is much improved or very much improved. ^*∗*^Statistically significant change from the baseline by visit in both treatment groups.

Analysis of PGIC scale showed a significant reduction over time by visit and dose, in both treatment groups, *P* ≤ 0.0001. Regarding the question of visit 5 “From study start, my health has improved a lot or a lot more” no difference was observed between both treatment groups (see Figure 3).

### 4.4. Adverse Events

Adverse events (AE) occurred in 44% of patients with pregabalin and 43% of patients with gabapentin/B1-B12. With PGB, the most common AE were dizziness (24%), somnolence (23%), headache (3%), and vertigo (4%); for the GBP/B1/B12 group they were dizziness (17%), somnolence (27%), light-headedness (24.1%), headache (7.5%), and vertigo (3.2%). Vertigo was less common in the GBP/B1-B12 group, with a statistically significant difference (*P* = 0.014). Comparing adverse events by dose used, 11% presented dizziness in the PGB group (300 mg/d) and 3% in the GBP group, with doses of 1800 mg/d, and a statistically significant difference (*P* = 0.0206).

## 5. Discussion

Our results show that the GBP plus vitamins B1-B12 combination is as effective as PGB for pain treatment. Pain intensity reduction was achieved with a 300 to 1800 mg/day dose of GBP/B1/B12 and in the same proportion as PGB 600 mg (maximum dose). Gorson et al. showed in a crossover study that GBP 900 mg per day is ineffective or minimally effective for PDN treatment [16]. Gómez-Pérez et al., in a parallel group trial, concluded that gabapentin doses greater than 1200 mg caused pain reduction in more than 50% [17] and Backonja and Glanzman showed, in a systematic review, that GBP doses (1800–3600 mg/d) are effective and safe for treating neuropathic pain [18]. It is possible that adding vitamins B1-B12 to GBP creates a synergistic effect, due to their antiallodynic and antihyperalgesic effect. The use of B vitamins for the treatment of PDN is controversial. Ang et al. reported in a meta-analysis that there is no sufficient evidence to recommend or disqualify the use of B complex vitamins when treating diabetic neuropathy, due to study heterogeneity [19].

Several mechanisms of action have been proposed to explain the effect of thiamine (B1) and cyanocobalamin (B12) when treating pain. Reyes-García et al. showed the synergy of GBP/B1-B12 as consequence of multiple effects of these vitamins at a metabolic level [6]. These effects can be divided into two categories, those decreasing damage mechanisms on nervous fibers and those with antihyperalgesic and antinociceptive effects [5].

Vitamin B1 decreases formation of protein glycation final products, which is a powerful generator of free radicals and oxidative stress [20]. Another effect is through inhibition of the diacylglycerol (DAG) pathway, which decreases protein kinase C (PKC) activation, thus decreasing damage to vascular endothelium. Likewise, it also reduces the activity of the hexosamine pathway; and it is through alternative pathways of the latter that metabolism improves via the pentose-phosphate pathway. B1 (thiamine diphosphate) works as coenzyme for erythrocyte transketolase, an essential enzyme for the metabolism of carbohydrates [21].

Clinical trials showed that 17–79% of type 1 and type 2 diabetics have thiamine deficiency, due to its participation in carbohydrate metabolism, with both euglycemic and hyperglycemic status [21]. The principal action of these effects is reducing nervous fiber damage, which is undoubtedly one of the factors contributing to the development of painful diabetic neuropathy.

B complex vitamins also act directly on pain control, since they have antiallodynic, antinociceptive, and antihyperalgesic effects [6]. Through the Nitric Oxide-Cyclic Guanosine Monophosphate pathway (NO-cGMP pathway), it potentiates soluble guanylyl cyclase and generates cGMP, while activating a type-G protein kinase (PGK), subsequently hyperpolarizing nociceptor potassium channels [22]. Likewise, it also increases nociceptive inhibitory control in afferent neurons of the spinal cord and reduces thalamic neuron response to nociceptive stimulation [23]. Another effect explaining its antihyperalgesic action is through an increase in serotonin and GABA synthesis, decreasing glutamate levels in several brain areas [24]. Therefore, the sum of all effects on carbohydrate metabolism and pain pathways explains its effectiveness in painful diabetic neuropathy.

Our study, through an improvement analysis measuring Patients' Global Impression of Change (PGIC), showed that both drugs improve pain. Backonja et al. showed, in clinical trials with PDN patients, efficacy of gabapentin in treating PDN with a moderate improvement of 60% in PGIC [9].

Both drugs increased sleep time from baseline visit. Backonja et al. showed doses of 1800 mg/d improved measurements in sleep interference scales [9, 18]. Lo et al. reported that GBP increases slow-wave sleep in primary insomnia patients, improving sleep quality (by increasing its efficiency and decreasing spontaneous awakening) [25].

Use of neuromodulators is associated with appearance of adverse events, particularly dizziness, vertigo, and somnolence, which in occasions limit use of greater doses and frequently cause treatment discontinuation. Freeman et al. showed, in a meta-analysis, that adverse events related to pregabalin use are dose-related, dizziness being the most common AE (28%), with 600 mg/d, followed by peripheral edema (16%) and somnolence (13%) [10]. The most frequently reported AE with GBP was dizziness (24%), somnolence (23%), and headache (11%) [9]. A safety and tolerability trial in 336 PDN patients showed reduced dizziness and vertigo frequency in the GBP/B1-B12 group versus pregabalin (*P* = 0.012 and *P* = 0.006, resp.) [5].

Decreased vertigo was observed with GBP/B1/B12 (GBP: 300 to 1800 mg, B1: 100 to 600 mg, and B12: 0.20 mg to 0.120 mg per day), compared to PGB (75–600 mg per day), *P* = 0.014, possibly related to the smaller GBP dose used in the study. The latter, in addition to the synergistic effect of vitamins B1 and B12, allowed for reduction of GBP dose needed to decrease pain intensity, achieving a greater safety and tolerability margin. Through a per dose analysis, less dizziness (3.4%) was observed with a 1800 mg dose in the GBP/B1-B12 group, compared to PGB 300 mg (11%), with a statistically significant difference, *P* = 0.0206.

## 6. Conclusion

One of this trial's strengths is that it shows that vitamins B1 and B12 have a synergistic effect in combination with gabapentin in PDN treatment, since pain intensity reduction was obtained with 50% of the GBP dose required as monotherapy. Likewise, regarding GBP dose reduction, there are less adverse events (vertigo). Nonetheless, it is necessary to confirm the role of vitamins, isolated and versus placebo, to prove the absolute and potential benefit of this combination.

## Figures and Tables

**Table 1:** Demographics characteristics and per group treatment characteristics (*n* = 270).

Demographics characteristics and per group treatment baselines, per protocol population					
	Pregabalin		Combined gabapentin		*P* value
	*n* = 123	%	*n* = 147	%	
Gender					
Female	74	60.2 95% CI (51.4–68.9)	100	68.0 95% CI (60.4–75.7)	0.179
Male	49	39.8 95% CI (31.1–48.6)	47	32.0 95% CI (24.3–39.6)	
Age (years)					
Average	53.6		52.5		0.344
Std. deviation	9.4		10.5		
Minimum–maximum	25.0–71.0		19.0–70.0		

*Risk factors*					

Smoking					
Yes	8	6.5 95% CI (2.1–10.9)	13	8.8 95% CI (4.2–13.5)	0.475
Arterial hypertension					
History	42	34.1 95% CI (25.6–42.6)	58	39.4 95% CI (31.5–47.4)	0.369
Hypothyroidism					
History	1	0.8 95% CI (0.0–2.4)	1	0.7 95% CI (0.0–2.0)	0.900
Cholesterol (baseline measure)					
Average	198.3		195.2		0.560
Std. deviation	47.5		38.3		
Minimum–maximum	101.0–542.0		71.0–366.0		
Triglycerides (baseline measure)					
Average	207.7		189.9		0.352
Std. deviation	183.4		116.3		
Minimum–maximum	53.0–1390.0		63.0–952.0		
BMI (Kg/m^2^)					
Average	28.2		27.9		0.610
Std. deviation	3.9		4.1		
Minimum–maximum	18.4–39.4		17.4–39.4		
Diabetes duration					
Average	9.8		9.5		0.765
Std. deviation	5.9		6.5		
Minimum–maximum	1.5–25.5		1.4–32.0		
With diabetic neuropathic					
Average	2.9		2.8		0.603
Std. deviation	1.1		1.1		
Minimum–maximum	0.6–5.9		1.0–5.9		
Diabetes treatment					
Oral	100	81.3 95% CI (74.3–88.3)	112	76.2 95% CI (69.2–83.2)	
Insulin	3	2.4 95% CI (0.0–5.2)	5	3.4 95% CI (0.4–6.2)	0.333
Both	20	16.3 95% CI (9.7–22.9)	30	20.4 95% CI (13.8–27.0)	
Glucose (baseline)					
Average	126.9		128.9		0.603
Std. deviation	53.0		51.2		
Minimum–maximum	44.0–410.0		64.0–325.0		
HbA1c					
Average	7.4		7.4		0.603
Std. deviation	1.3		1.4		
Minimum–maximum	5.2–10.2		4.9–10.0		
